# Mutations in voltage-gated L-type calcium channel: implications in cardiac arrhythmia

**DOI:** 10.1080/19336950.2018.1499368

**Published:** 2018-08-15

**Authors:** Qing Zhang, Junjie Chen, Yao Qin, Juejin Wang, Lei Zhou

**Affiliations:** a Department of Cardiology, the Second Affiliated Hospital of Nantong University, Nantong First Hospital, Nantong, Jiangsu, China; b Department of Cardiology, the First Affiliated Hospital of Nanjing Medical University, Nanjing, Jiangsu, China; c Department of Physiology, Nanjing Medical University, Nanjing, Jiangsu, China

**Keywords:** Cardiac arrhythmia, L-type calcium channel, mutation

## Abstract

The voltage-gated L-type calcium channel (LTCC) is essential for multiple cellular processes. In the heart, calcium influx through LTCC plays an important role in cardiac electrical excitation. Mutations in LTCC genes, including *CACNA1C, CACNA1D, CACNB2* and *CACNA2D*, will induce the dysfunctions of calcium channels, which result in the abnormal excitations of cardiomyocytes, and finally lead to cardiac arrhythmias. Nevertheless, the newly found mutations in LTCC and their functions are continuously being elucidated. This review summarizes recent findings on the mutations of LTCC, which are associated with long QT syndromes, Timothy syndromes, Brugada syndromes, short QT syndromes, and some other cardiac arrhythmias. Indeed, we describe the gain/loss-of-functions of these mutations in LTCC, which can give an explanation for the phenotypes of cardiac arrhythmias. Moreover, we present several challenges in the field at present, and propose some diagnostic or therapeutic approaches to these mutation-associated cardiac diseases in the future.

## Introduction

Cardiac arrhythmia is one of the major causes for sudden cardiac death (SCD) [1,2]. Over the past decades, some arrhythmic susceptible genes, including voltage-gated L-type calcium channel (LTCC), have been identified many arrhythmia-associated mutations [3–5]. LTCC is the main pathway for calcium ion influx into excitable cells in response to the membrane depolarization [6,7], which forms one part of cardiomyocyte action potentials (APs). In the heart, LTCC is a multi-subunit protein complex composed by four subunits: α_1_ subunit and auxiliary β, α_2_δ and γ subunits, encoded by *CACNA1C* or *CACNA1D, CACNB2, CACNA2D* and *CACNG*, respectively [8,9]. It has been known that the mutations in these LTCC genes induce the dysfunctions of calcium channels, which result in the abnormal excitations of cardiomyocytes, and finally lead to cardiac arrhythmias.

**Figure 1.**
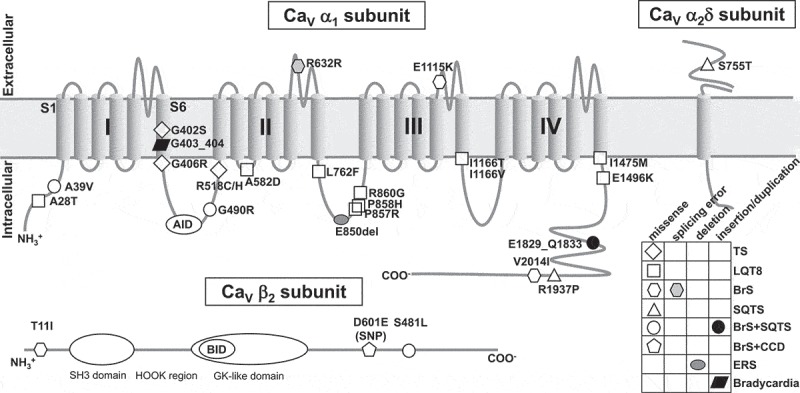
Predicted topology of Ca_V_ α_1_ subunit with associated β_2_ and α_2_δ subunits shows the location of functional mutations. All mutations in α_1_ subunit are derived from α_1C_ (Ca_V_1.2), except one mutation G403_404ins is come from α_1D_ (Ca_V_1.3). AID = α-subunit interaction domain; BID = β-subunit interaction domain; BrS = Brugada syndrome; CCD = cardiac conduction disease; ERS = early repolarization syndrome; GK = guanylate kinase; LQTS = long QT syndrome; SH3 = Src homology 3; SQTS = short QT syndrome; SNP = single nucleotide polymorphism; TS = Timothy syndrome.

In this work, we summarize recent findings on the mutations in the different subunits of LTCC, which are associated with cardiac arrhythmias (Figure 1), and describe the functional roles of these mutations in channel properties (Table 1), which can shed a light in understanding of cardiac electrophysiological characteristics in these diseases.

**Table 1.** Summary of mutations or polymorphisms in *CACNA1C, CACNA1D, CACNB2b* and *CACNA2D1.*

No.	Amino acid change	Nucleotide change	Exon	Location	Mutation type	Gain/loss of function	Main effects on LTCC	Diagnosis	References
Mutations in *CACNA1C*									
1	p.A28T	c.82 G > A	2	N-terminus	Missense	Gain	*I*_Ca,L_ ↑, positively shift of *V*_0.5,inact_	LQT8	Wemhoner et al. 2015^82^
2	p.A39V	c.116 C > T	2	N-terminus	Missense	Loss	*I*_Ca,L_ ↓, LTCC trafficking ↓	BrS3/SQT4	Antzelevitch et al. 2007^98^
3	p.P381S	c.1141 C > T	8A	DI-S6	Missense	-	N.S.	LQT8	Fukuyama et al. 2014^88^
4	p.G402S p.G406R	c.1204 G > A c.1216 G > A	8	DI-S6	Missense	Gain	VDI ↓	TS2	Splawski et al. 2005^62^
5	p.G406R	c.1216 G > A	8A	DI-S6	Missense	Gain	VDI ↓↓, CDI ↑	TS1	Splawski et al. 2004^61^; Barrett et al. 2008^66^
6	p.M456I	c.1368 G > A	9	I-II loop	Missense	-	N.S.	LQT8	Fukuyama et al. 2014^88^
7	p.G490R	c.1468 G > A	10	I-II loop	Missense	Loss	*I*_Ca,L_ ↓	BrS3/SQT4	Antzelevitch et al. 2007^98^
8	p.R518C/H	c.1552 C > T/ c.1553 G > A	12	I-II loop	Missense	Loss/gain	*I*_Ca,L_ ↓, Ca_V_1.2 inactivation ↓, window current ↑, late current ↑	Cardiac only TS	Boczek et al. 2015^83^
9	p.A582D	c.1745 C > A	13	DII-S2/S3	Missense	Gain	Ca_V_1.2 inactivation ↓	LQT8	Fukuyama et al. 2014^88^
10	p.R632R	c.1896 G > A	14	DII-S5/S6	Splicing error	Loss	LTCC mRNA ↓	BrS3	Fukuyama et al. 2014^101^
11	p.L762F	c.2284 C > T	16	II-III loop	Missense	Gain	Ca_V_1.2 inactivation ↓, window current ↑	LQT8	Landstrom et al. 2016^89^
12	p.E850del	c.2548_2550 delGAG	19	II-III loop	Deletion	Loss	*I*_Ca,L_ ↓↓	ERS2	Burashnikov et al. 2010^102^ Sutphin et al, 2016^90^
13	p.P857R	c.2570 C > G	19	II-III loop	Missense	Gain	*I*_Ca,L_ ↑, surface expression ↑	LQT8	Boczek et al. 2013^84^
14	p.R858H	c.2573 G > A	19	II-III loop	Missense	Gain	*I*_Ca,L_ ↑	LQT8	Fukuyama et al. 2014^88^
15	p.R860G	c.2578 C > G	19	II-III loop	Missense	Gain	SSC ↑, positively shift of *V*_0.5,inact_	LQT8	Wemhoner et al. 2015^82^
16	p.E1115K	c.3343 G > A	26	DIII-S5/S6	Missense	Loss	Single channel conductance ↓	BrS3	Burashnikov et al. 2010^102^ Simms. 2014^103^
17	p.I1166V	c.3496 A > G	28	DIII-S6	Missense	Gain	*I*_Ca,L_ ↑	LQT8	Wemhoner et al. 2015^82^
18	p.I1166T	c.3497 T > C	28	DIII-S6	Missense	Gain	*I*_Ca,L_ ↑, negatively shift of *V*_0.5,act_	LQT8	Boczek et al. 2015^81^ Wemhoner et al. 2015^82^
19	p.I1475M	c.4425 C > G	38	DIV-S6	Missense	Gain	SSC ↑, Negatively shift of *V*_0.5,act_	LQT8	Wemhoner et al. 2015^82^
20	p.E1496K	c.4486 G > A	38	C-terminus	Missense	Gain	Negatively shift of *V*_0.5,act_, SSC ↑, current decay ↓	LQT8	Wemhoner et al. 2015^82^
21	p.G1783C	c.5347 G > T	44	C-terminus	Missense	-	N.S.	LQT8	Fukuyama et al. 2014^88^
22	p.E1829_Q1833dup	c.5485_5499 dup15	43	C-terminus	Duplication	Loss	*I*_Ca,L_ ↓↓	BrS3/SQT4	Burashnikov et al. 2010^102^
23	p.R1937P	c.5918 G > C	46	C-terminus	Missense	Loss	*I*_Ca,L_ ↓↓, negatively shift of *V*_0.5,inact_	SQT4	Chen et al. 2017^116^
24	p.V2014I	c.6040 G > A	46	C-terminus	Missense	Loss	*I*_Ca,L_ ↓, negatively shift of *V*_0.5,inact_	BrS3	Burashnikov et al. 2010^102^
25	p.N2091S	c.6272A> G	47	C-terminus	Missense	Gain	*I*_Ca,L_ ↑, negatively shift of *V*_0.5,act_	-	Sutphin et al, 2016^90^
26	p.K834E	c.2500 A > G	19	II-III loop	Missense	Unknown	Unknown	LQT8	Boczek et al. 2013^84^
27	p.P857L	c.2570 C > T	19	II-III loop	Missense	Unknown	Unknown	LQT8	Boczek et al. 2013^84^
28	p.A1473G	c.4418 C > G	38	DIV-S6	Missense	Unknown	Unknown	TS	Gillis et al. 2012^80^
29	p.R1880Q	c.5639 G > A	44	C-terminus	Missense	Unknown	Unknown	BrS3	Burashnikov et al. 2010^102^
30	p.C1873Y	c.5510 G > A	45	C-terminus	Missense	Unknown	Unknown	BrS3/SQT4	Burashnikov et al. 2010^102^
31	p.R1906Q	c.5717 G > A	44	C-terminus	Missense	Unknown	Unknown	LQT8	Boczek et al. 2013^84^
32	p.R1977Q	c.6167 G > A	47	C-terminus	Missense	Unknown	Unknown	SQT4	Mazzanti et al. 2014^115^
33	p.D2130N	c.6388 G > A	47	C-terminus	Missense	Unknown	Unknown	BrS3	Burashnikov et al. 2010^102^
Mutations in *CACNA1D*									
1	p.G403_404ins	c.1028_1029 insGGG	8B	DI-S6	Insertion	Loss	Conduction of Ca_V_1.3 ↓	Bradycardia	Baig et al. 2011^126^
Mutations in *CACNB2b*									
1	p.T11I	c.32 C > T	2	N-terminus	Missense	Loss	Fast and slow decay ↑	BrS4	Cordeiro et al. 2009^104^
2	p.S481L	c.1442 C > T	14	C-terminus	Missense	Loss	*I*_Ca,L_ ↓	BrS4/SQT5	Antzelevitch et al. 2007^98^
3	p.D601E	c.1803 T > G (polymorphism)	13b	C-terminus	Missense	Gain	Late *I*_Ca,L_ ↑, Ca_V_1.2 inactivation ↓	BrS4/CCD	Burashnikov et al. 2010^102^ Hu et al. 2010^119^
4	p.A73V	c.218 C > T	4	SH3 domain	Missense	Unknown	Unknown	IVF	Burashnikov et al. 2010^102^
5	p.S143F	c.428 C > T	5	HOOK region	Missense	Unknown	Unknown	BrS4	Burashnikov et al. 2010^102^ Risgaard et al. 2013^107^
6	p.S160T	c.479 G > C	6	HOOK region	Missense	Unknown	Unknown	BrS4	Burashnikov et al. 2010^102^
7	p.K170N	c.510 G > T	7b	HOOK region	Missense	Unknown	Unknown	BrS4/CCD	Burashnikov et al. 2010^102^ Kanter et al. 2012^110^
8	p.L399F	c.1195 C > T	12	GK-like domain	Missense	Unknown	Unknown	BrS4/CCD	Burashnikov et al. 2010^95^; Kanter et al. 2012^110^
9	p.T450I	c.1349 C > T	14	C-terminus	Missense	Unknown	Unknown	BrS4	Burashnikov et al. 2010^95^; Risgaard et al. 2013^107^
10	p.D538E	c.1614 C > A	13	C-terminus	Missense	Unknown	Unknown	BrS4/CCD	Kanter et al. 2012^118^
11	p.R571C	c.1711 C > T	14	C-terminus	Missense	Unknown	Unknown	BrS4	Burashnikov et al. 2010^102^
Mutations in *CACNA2D1*									
1	p.S755T	c.2264 G > C	28	Extracellular	Missense	Loss	*I*_Ca,L_ ↓	SQT6	Templin et al. 2011^117^
2	p.D550Y	c.1648 G > T	19	Cache domain	Missense	Unknown	Unknown	BrS9	Burashnikov et al. 2010^102^
3	p.S709N	c.2126 G > A	26	Extracellular	Missense	Unknown	Unknown	BrS9	Burashnikov et al. 2010^102^ Risgaard et al. 2013^107^
4	p.Q917H	c.2751 A > T	34	Extracellular	Missense	Unknown	Unknown	BrS9	Burashnikov et al. 2010^102^ Risgaard et al. 2013^107^
5	p.S956T	c.2867 C > A	36	Extracellular	Missense	Unknown	Unknown	ERS4	Burashnikov et al. 2010^102^

BrS = Brugada syndrome; CDI = calcium-dependent inactivation; CCD = cardiac conduction disease; ERS = early repolarization syndrome; *I*_Ca,L_ = calcium currents of L-type channel; IVF = idiopathic ventricular fibrillation; LQT = long-QT syndrome; LTCC = L-type calcium channel; SQT = short-QT syndrome; SSC = steady-state current; TS = Timothy syndrome; *V*_0.5,act_ = half-activation potential; *V*_0.5,inact_ = half-inactivation potential; VDI = voltage-dependent inactivation.

N.S. = no significant differences vs. wild type channel.

## Molecular basis of L-type calcium channel

The pore-forming α_1_ subunit, encoded by *CACNA1C* or *CACNA1D* gene, is the dominant voltage-gated calcium channels expressed in the working cardiomyocytes or sinoatrial nodal cells (SANCs), respectively. This α_1_ subunit determines the main pharmacologic and biophysical properties of the channel [8,10], it contains four repeated homologous domains (DI-DIV), and each domain is comprised of six transmembrane segments (S1-S6) (Figure 1) [10]. Ca_V_1.2 α_1C_ (*CACNA1C*) plays a critical role in the cardiovascular function. Deletion of α_1C_ subunit in mice resulted in embryonic lethality [11], and conditional knockout of smooth muscle α_1C_ (SMAKO) lowered the arterial blood pressure in mice [12]. Recently, multiply alternative splicing events have been found in *CACNA1C*, which optimize the functions of Ca_V_1.2 channel [13,14]. Moreover, alternative splicing in Ca_V_1.2 channels make some roles in several cardiovascular diseases, including cardiac arrhythmia [15–17]. Ca_V_1.3 α_1D_ (*CACNA1D*) is highly expressed in both SANCs and cochlear inner hair cells [18–21]. Targeted deletion of α_1D_ caused deafness, pronounced bradycardia, and nonfatal sinoatrial arrhythmia in mice [22,23].

The auxiliary β subunit modifies the gating property of α_1_ subunit, it can increase the calcium currents by regulating the expression of α_1_ subunit in the cell membrane [6,24,25]. Mechanistically, Ca_V_β works as a chaperone for the α subunit, and its β-interaction domain (BID) binds with the reserved α-interaction domain (AID) located at I-II loop of Ca_V_α_1_ subunit, enhancing the trafficking of the channels from endoplasmic/sarcoplasmic reticulum (ER/SR) to cell membrane [24,26]. To date, 4 genes encoding β subunits (β_1-4_) have been identified [27,28]; of which, Ca_V_β_2_ subunit, encoded by *CACNB2* gene, is the dominant variant expressed in the heart [29], which has at least 8 distinct splice variants (β_2a-h_) [30,31].

The extracellular α_2_ and transmembrane δ subunit, linked with each other via disulfide bonds, are encoded by the gene *CACNA2D* [32]. The α_2_δ-1 subunit, encoded by *CACNA2D1*, is abundant in skeletal and cardiac muscles [33,34]. The α_2_δ-2 and α_2_δ-3 subunits, encoded by *CACNA2D2* and *CACNA2D3* respectively, express in neurons and some other tissues [33,35]. In addition, the expression of α_2_δ-4 subunit, encoded by C*ACNA2D4*, is mostly copious in non-neuronal cells apart from retinal neurons [36,37]. By bioinformatics, the proteins encoded by several other genes have been identified, and they share the similar structure with α_2_δ subunits [38], but have not been proved to function as calcium α_2_δ subunits. The α_2_δ-1 subunit plays a vital role in hypertensive vascular Ca_V_1.2 channel properties [39,40]. In the heart, the atrium is characterized by increased α_2_δ-1 protein expression as compared with the ventricle, which might lead to the Ca_V_1.2 electrophysiological differentiation between atrial and ventricular cardiomyocytes [41]. Furthermore, deletion of α_2_δ-1 in mice induced the decreased Ca_V_1.2 Ca^2+^ currents of cardiomyocytes, and also decreased the basal myocardial contractility and relaxation [42]. These observations imply the potential roles of α_2_δ subunits in cardiac arrhythmias.

The γ subunit, which is encoded by *CACNG*, has 8 isoforms and consists of 4 transmembrane domains [43]. The first γ_1_ subunit was cloned from skeletal muscle [44,45], whereas it was not expressed in cardiac muscle [46]. The γ subunit distinguishingly modulates the functions by altering both activation and inactivation properties of LTCC [46]. For example, targeted disruption of γ_1_ subunit increased the amplitude of peak calcium current in isolated myotubes [47]; whereas coexpression with γ_2_ subunit could depolarize the activation and inactivation curve of Ca_V_1.2 channels [48]. In the heart tissue, four different γ subunits, γ_4_, γ_6_, γ_7_ and γ_8_, are expressed, and they all physically interact with the Ca_V_1.2 complex, which diversify the functions of Ca_V_1.2 channels [46]. To date, no evidences indicate the association of γ subunits and cardiac arrhythmias, thus it is of value to investigate their possible links.

## Long QT and Timothy syndromes

Long QT syndrome (LQTS) is one of the most common cardiac electrophysiological diseases with an estimated prevalence of 1 in every 2,534 persons [49]. It is a typical cardiac repolarization abnormality defined by heart rate-corrected QT interval (QTc) prolongation on resting electrocardiogram (ECG) [4,50], and characterized by an increased trend for ventricular tachycardia with *torsade de pointes* (TdP). LQTS exists as a congenital genetic disease (cLQTS) with mutations described in different genes, which cause different types of LQTS. LQTS also can be acquired (aLQTS) by drug-intake [51] or structural heart disease [52], which may be more prevalent than cLQTS [53]. To date, 16 genes were identified to responsible for LQTS, of which, mutations in *CACNA1C* is accounted for long QT syndrome 8 (LQT8) [54]. Two canonical mutations in *CACNA1C* (p.G406R and p.G402S) are associated with a severe LQT8, namely Timothy syndrome (TS).

### Timothy syndrome 1

In the 1990s, a severe syndrome which was called as the heart-hand syndrome, now known as TS or TS1, was observed in young children with significant clinical phenotypes: syndactyly, severe cardiac arrhythmia, congenital heart disease, developmental abnormalities, autism, and neurological dysfunction [55–57]. Most patients were diagnosed during neonatal period or rarely in late infancy, only two cases reported the fetal hydrops as antenatal expression of TS1 [58,59].

Splawski et al. discovered a *de novo* G-to-A mutation, which caused a p.G406R substitution in translational sequence at position 1216 (c.G1216A) of *CACNA1C* exon 8A (also known as exon 8 [60]) in all 13 individuals with TS. However, the p.G406R mutation was not identified in 180 ethnically matched controls [61], indicating that p.G406R mutation is associated with TS. This amino acid p.G406 is completely conserved in Ca_V_α_1C_ subunit of other mammalian species, and is located at the C-terminal portion of the S6 of domain I [61,62].

The inactivation of the voltage-gated calcium channel has two mechanisms: voltage-dependent inactivation (VDI) and calcium-dependent inactivation (CDI), and they play an essential role in controlling excitation-contraction coupling in cardiomyocytes [6,63–65]. Functional analysis revealed that p.G406R mutation caused the maintained inward calcium currents by impairing the two types of channel inactivation [61]. By measuring whole-cell ionic currents in transfected human embryonic kidney-293 (HEK293) cells, the p.G406R mutation slowed the VDI of Ca_V_1.2 calcium channels, while it possibly accelerated the kinetics of CDI [66]. It is known that the small glycine residues offer flexibility to allow for the I-II loop to interact with the intracellular pore of the channel [67–69], and G406 is located wiαthin a short stretch of nonhelical motif at the start of the α_1C_ I-II loop [70]. Therefore, p.G406R mutation would produce a more bulky arginine residue to impede the movement of the I-II loop, leading to the slower channel inactivation. As a result of abnormally huge calcium ion influx, the action potential duration (APD) can be prolonged. Thus, the patients’ ECG showed a prolonged QT interval leading to death from cardiac arrhythmia [66]. Interestingly, this mutant Ca_V_1.2 channel function could be modulated by one anchoring protein AKAP150, elimination of which could abrogate the prolonged QT interval [71], implying AKAP150 may be a promising target for TS.

To explore the effects of the p.G406R mutation on the electrical activity and contraction of living cardiomyocytes, human skin cells from TS patients were reprogrammed to generate induced pluripotent stem cells (iPSC), and differentiated into cardiomyocytes [72]. The electrophysiological recording and calcium imaging studies revealed excessive calcium ion influx, irregular electrical activity and contraction, prolonged APs, and abnormal calcium transients in these ventricular-like cells [72,73]. Roscovitine, both an atypical LTCC blocker and a cyclin-dependent kinase inhibitor, which can enhance the VDI of Ca_V_1.2, restored the electrical and calcium signaling properties of cardiomyocyte from TS patients [72–75]. Recently, TS mouse model had been generated and proved to have more arrhythmogenic events, which might be due to the increase of cytosol calcium concentration ([Ca^2+^]_i_) by sarcolemmal Ca^2+^ leak and the impairment of VDI [76]. Syndactyly or craniofacial abnormalities may also be caused by irregular Ca_V_1.2^TS^ channels, because Ca_V_1.2^TS^ channels affected the development of jaw and mandibular in the mouse and zebrafish models [77].

### Timothy syndrome 2

Splawski et al. also identified two mutations, c.G1204A and c.G1216A, in exon 8 (also known as exon 8a [60]) of the *CACNA1C* gene, resulting in the p.G402S and p.G406R substitution, respectively [62]. Owing to that the exon 8 splice variant was found to highly express in human heart and brain, the patients who carried this mutation had a longer QT interval and more severe arrhythmia than patients with a mutation in exon 8A. Since the symptoms caused by the mutations in exon 8 were different from previous TS, this disease was named as TS2. The patients suffered from multiple arrhythmias and SCD due to the extreme prolongation of QT interval, but syndactyly might not be manifested [62,78], and these patients were more likely died of TdP and ventricular fibrillation [62]. Structurally, the glycine residue at 402 position of IS6, together with the same positions in the IIS6, IIIS6 and IVS6 segments, form the G/A/G/A motif, which is near the inner channel mouth of Ca_V_1.2. These four residues interact with larger bulky residue from neighboring S6 helices to stabilize the inactivation gate [68]. In TS2, in addition to p.G406R mutation, p.G402S will also introduce a more bulky serine residue to disrupt the tightly sealing of the S6 helices, resulting in the slower deactivation [68]. Although neither heterozygous nor homozygous p.G402S transgenic mice can survive to weaning because of the high expression of exon 8 and a fatal extremely high level of calcium channels mutation, TS2-like mouse model with p.G406R had been generated by heterozygously keeping an inverted neomycin cassette in exon 8A [79], these mice could survive through adulthood due to the lowered expression of p.G406R LTCCs. The survived mice displayed the behavioral abnormalities, corresponding to the core aspects of autism spectrum disorder [79].

### Timothy syndrome with novel CACNA1C mutations

A newborn, with healthy parents, had the similar symptoms with TS, he presented with prolonged QT interval and associated polymorphic ventricular tachycardia, dysmorphic facial features, syndactyly of the hands and feet, and joint contractures. By full gene sequencing, the patient had no p.G402S and/or p.G406R mutations, but a novel *CACNA1C* mutation p.A1473G had been detected in exon 38 [80]. This case expanded the molecular basis of TS, however the electrophysiological properties of this mutant channel need further investigations. Recently, another novel *CACNA1C* mutation p.I1166T in exon 28 was identified in a young male with diagnosed TS, and this mutation induced an overall loss of current density and a shift of activation of Ca_V_1.2 channels, which lead to an increased window current [81]. However, Wemhoner et al. found this p.I1166T mutation could induce a leftward shift of activation curve, but an increased peak current density [82]. These different results maybe attribute to different clones of Ca_V_1.2 subunits, or even different ratios of subunits expressed in HEK293 cells.

### Long QT syndromes without extracardiac signs

Some LQT8, with typical long QT interval, have no extracardiac phenotypes. A novel mutation p.R518C/H in *CACNA1C* was just identified in patients with a complex phenotype including LQTS, hypertrophic cardiomyopathy and congenital heart defects, which was annotated as “cardiac only TS” [83]. This mutation in Ca_V_1.2 also revealed a complex channel phenotypes, including loss of current density and inactivation in combination with increased window and late currents [83]. By whole-exome sequencing and bioinformatics/systemic biology, Boczek et al. further identified 4 novel mutations of *CACNA1C* in LQTS, including p.K834E, p.P857R, p.P857L, and p.R1906Q [84], 3 of which were located at the conserved proline, glutamic acid, serine and threonine rich (PEST) domain of Ca_V_α_1_ II-III loop, this domain acts to proteolytic signaling through the cellular quality control system [85]. Functionally, p.P857R mutation, cosegregated with the disease within the pedigree, significantly increased calcium currents and surface membrane expression of the channel as compared with wild type (WT) Ca_V_1.2 channel [84]. Interestingly, p.R1906Q locates one amino acid away from α_1C_ C-terminal STIM1 (stromal-interacting molecule 1, a calcium store sensor) binding domain, which may affect STIM1-mediated Ca_V_1.2 channel gating inhibition and channel internalization [86,87]. The identification of these *CACNA1C* mutations co-segregating with disease indicated that *CACNA1C* genetic perturbations might underscore autosomal dominant LQT8 in the absence of TS.

Additionally, five novel *CACNA1C* mutations (p.P381S, p.M456I, p.A582D, p.R858H, and p.G1783C) were identified in the patients with LQT8, but without typical TS phenotypes [88]. Of significance, p.R858H mutant channel had a larger *I*_Ca_, and p.A582D mutant channel displayed a slower inactivation [88], which could partially explain the phenotype of long QT interval. By screening 540 patients with LQTS, 6 more *CACNA1C* mutations were identified recently, including p.A28T, p.R860G, p.I1166T, p.I1166V, p.I1475M and p.E1496K [82]. These mutations affected the different properties of channel. Briefly, p.A28T increased calcium current of L-type channel (*I*_Ca,L_) and positively shifted steady-state inactivation (SSI). p.R860G positively shifted SSI and increased steady-state current (SSC). Both p.I1166T and p.I1166V mutations showed an increased *I*_Ca,L_, but p.I1166T negatively shifted the steady-state activation (SSA) and increased SSC. p.I1475M and p.E1496K negatively shifted the SSA, moreover p.E1496K increased SSC and slowed the current decay [82]. Using computational cardiac AP model, the researchers found these gain-of-function mutations delayed repolarization of the cardiac myocytes, which induced prolongation of APD [82]. Recently, a novel *CACNA1C* mutation p.L762F was identified to associate with the development of LQTS. This mutation slowed the channel inactivation and increased persistent and window current, which attributed to the gain-of-function of Ca_V_1.2 channels [89]. Furthermore, a novel mutation of *CACNA1C* (p.N2091S) is also found in autopsy-negative sudden unexplained death of a 24-year-old white female. No premortem ECGs were available and neither decedent had any documented family history of arrhythmia-related cardiac events. However, this p.N2091S mutation induced a dramatic increase of *I*_Ca_ and a minor hyperpolarization of *V*_0.5_ of channel activation, indicating a LQTS-like gain-of-function electrophysiological phenotype [90].

## Brugada syndromes

Brugada syndrome (BrS), first described in 1992, is an inherited cardiac arrhythmic syndrome associated with a high risk of ventricular fibrillation without structural heart disease [91]. The ECG of the Brugada patient is characterized by right bundle branch block and ST segment elevation in precordial leads V1-V3.

The symptom of BrS typically manifests during the adulthood, but the youngest individual diagnosed with BrS is only 2 days old, while the oldest is 84 years old [92,93]. Generally, BrS is more prevalent among male patients owing to the gender differences in the expression of cardiac transient outward potassium channel (*I*_to_). Because of the presence of a more predominant *I*_to_ in males compared with females, male patients are more likely to induce the loss of the AP dome and the development of phase 2 reentry and polymorphic ventricular tachycardia [94,95]. Mutations in *CACNA1C, CACNB2* and *CACNA2D1* can cause BrS3, BrS4 and BrS9, respectively [96,97].

### Brugada syndrome 3

A male European descent with BrS and a shortened QTc interval showed a heterozygous mutation in exon 2 of the *CACNA1C* gene, which made a p.A39V substitution, near the N-terminus within a highly conserved region of the Ca_V_α_1C_ [98]. In another case, a male Turkish patient with BrS and a shortened QT interval, was detected a novel heterozygous mutation in exon 10 of the *CACNA1C* gene was predicted to result in a p.G490R substitution in I-II loop [98]. Patch-clamp analysis in Chinese hamster ovary (CHO) cells, which transfected with p.A39V or p.G490R mutant Ca_V_1.2 channels, showed the current amplitude was dramatically reduced as compared with WT channels, although voltage at peak current remained unchanged [98]. These results gave an sound explanation for the shortened QT interval. Moreover, p.A39V mutant channel had a defect in trafficking of mature LTCCs, however, confocal imaging revealed the normal trafficking of p.G490R mutant Ca_V_1.2 channels from ER to cell membrane [98]. Therefore, it is reasonable to conclude that p.G490R mutation may affect the channel open probability (P_o_), but not cell surface expression. Interestingly, this p.A39V mutation in N-terminus also affected the calmodulin-dependent activation of Ca_V_1.2 channel [99], but didn’t affect the surface expression of neuronal Ca_V_1.2 channels [100], which might be involved the mechanisms of BrS. Recently, a p.R632R in Ca_V_1.2 α_1C_ due to c.G1869A mutation in exon 14 of *CACNA1C* gene was identified in a patient with BrS, this mutation caused an aberrant splicing, which in turn made a premature stop codon in the downstream. This type of mutation might lead to nonsense-mediated mRNA decay, which induced the loss-of-function of Ca_V_1.2 calcium channel [101].

By screening 162 probands with BrS or BrS combined with short QT syndrome (SQTS) (BrS+ SQTS), there were 7 newly identified mutations in *CACNA1C*, including p.E1115K, p.R1880Q, p.V2014I, p.D2130N, p.E1829_Q1833dup, p.C1873Y and p.E850del [102]. Functionally, Ca_V_1.2 channel with p.E1115K had a reduced calcium influx. Mechanistically, this mutation strictly reduced single channel conductance, but did not change the voltage or calcium-dependent gating [103]. p.V2014I mutation in Ca_V_1.2 channel reduced peak current density and shifted half-inactivation voltage to more negative potentials, whereas voltage at the maximum peak current remained unchanged. In another mutation, the duplication of five amino acids in exon 43 of *CACNA1C* (p.E1829_Q1833dup) resulted in nearly compete suppression of calcium current (*I*_Ca_) [102]. These loss-of-function mutations may help to explain the shortening of QT interval and other features of ECG.

### Brugada syndrome 4

In two right precordial ECG leads, the patient displayed a type I ST-segment elevation, and a novel p.T11I mutation in *CACNB2b* was identified. This mutant Ca_V_β_2b_ had a faster fast and slow decay *I*_Ca_ and a reduced total charge compared to WT Ca_V_β_2b_, but had almost same peak calcium current density, SSI and recovery from inactivation [104]. In 6 affected family members with BrS and a shortened QTc interval, a c.C1442T heterozygous mutation in exon 13 of the *CACNB2b* gene resulted in p.S481L substitution downstream of the BID of β_2b_ subunit [98]. This mutation was not found in either 4 phenotype-negative family members or in 400 ethnically matched control alleles. The calcium current in the cells coexpressing with p.S481L Ca_V_β_2b_ was dramatically smaller than WT Ca_V_β_2b_, but the surface expression was almost the same, which indicated that the p.S481L channel traffics normally [98]. Due to the fact that the mutation is located at the position nearby the BID domain binding with α_1C_ subunits, the mechanism of dysfunctions of calcium channel could be interfered by the stimulatory role of β_2_ subunit on LTCC [105].

A patient with BrS, who has a p.V2014I mutation in *CACNA1C*, also had a p.D601E polymorphism in *CACNB2b*, and this polymorphism dramatically augmented the late calcium current of LTCC, which prolonged the QT interval, the modulatory effect of this single nucleotide polymorphism probably interprets the fact that QTc in this proband is not associated with SQTS [102]. Other mutations were also identified [102,106], including p.S143F and p.T450I, which were reported in the prevailing BrS-associated variants of NHLBI GO Exome Sequencing Project population [107]. However, the functions of these mutations on the Ca_V_β_2_ subunit remain unknown.

### Brugada syndrome 9

Human mutations in *CACNA2D1* have been identified to be associated with several kinds of cardiac dysfunctions, including BrS [97]. It has been found 3 different missense mutations in *CACNA2D1* (p.S709N, p.D550Y, and p.Q917H) in 3 BrS patients from a cohort made up of 205 patients with BrS [102]. Among these mutations, p.S709N and p.Q917H were also identified in Exome Sequencing Project population by Risgaard et al. [107]. Nevertheless, more works are needed to explore the possible functions of these mutations on Ca_V_ channels.

## Short QT syndromes

The short QT syndrome (SQTS), first described in 2000 [108], was defined by an abnormally shortened QT intervals and a propensity for cardiac arrest (CA) [109,110]. SQTS has been associated with the gain-of-function mutations in 3 distinct potassium channels, *KCNH2, KCNQ1* and *KCNJ2*, which cause SQT1, SQT2 and SQT3, respectively [111–114]. Nowadays, more evidences indicate the mutations in LTCC are also linked to SQTS. Some cardiac arrhythmias have the combined phenotypes of SQTS and BrS, since the shortened QT interval is also one of the manifestations of BrS. Thus, BrS3, caused by the mutation in *CACNA1C*, such as p.A39V, p.G490R, p.E1829_Q1833 duplication, and p.E850 deletion, also known as SQT4; and BrS4, caused by *CACNB2* p.S481L mutation, is also known as SQT5 [98].

### Short QT syndrome 4

One proband’s ECG showed that the QTc interval of the patient was shorter than that of healthy controls and characterized with aborted CA, and a novel *CACNA1C* mutation (p.R1977Q) was first identified in this SQTS patient, but the parents didn’t carry this mutation [115]. However, the roles of p.R1977Q mutation in SQTS are still unknown. Recently, a novel *CACNA1C* mutation p.R1937P was reported in a Chinese family of hypertrophic cardiomyopathy with early repolarization and SQTS, p.R1937P induced the loss-of-function of Ca_V_1.2 channels, which dramatically decreased the *I*_Ca_ and hyperpolarized the SSI [116].

### Short QT syndrome 6

A novel type of SQTS (SQT6) caused by a mutation p.S755T in the *CACNA2D1* was reported in 2011 [117]. The ECG of the patient revealed a shortened QT interval. Templin et al. took advantage to analyze the functional roles of the p.S755T mutation in transfected HEK293 cells. Compared with the WT variant, p.S755T α_2_δ-1 subunit extremely reduced the barium currents under the whole-cell patch clamp recording [117]. Western blotting showed that the membrane expression of α_1C_ subunit with mutant α_2_δ-1 was similar to WT α_2_δ-1 subunit, thus this mutation might only alter the open probability (*P*_o_) of the Ca_V_1.2 channels without modulating the membrane expression of the α_1C_ subunit of LTCC.

## Other cardiac arrhythmias

### Cardiac conduction disease

Combined mutations in *CACNB2b* (p.K170N/L399F) in one infant displaying BrS phenotype had a severe intraventricular conduction delay, and another case with conduction delay had identified p.D538E mutation in *CACNB2b* [118]. A novel polymorphism (p.D601E) was also identified in *CACNB2b* in the family displaying first-degree atrioventricular block, which induced the slowed inactivation of Ca_V_1.2 channels by strongly increasing the total charges [119]. Together with the mutation of *SCN5A* (Na_V_1.5 sodium channel) in this case, the sodium current reduction can cause loss of the right ventricular epicardial AP dome absent from the slowed inactivation of calcium current and slowed conduction, whereas p.D601E polymorphism could restore the dome of APs [119]. These results suggest the gain-of-function mutation in calcium channel can rescue the loss-of-function of sodium channel mutation in the process of cardiac conduction disease without BrS. It is notable that the p.D601E polymorphism in *CACNB2b* was also found in the BrS, and this polymorphism could increase the late calcium current [102], indicating the multiply functions of p.D601E polymorphism.

### Idiopathic ventricular fibrillation and early repolarization syndrome

Early repolarization has been found in patients with idiopathic ventricular fibrillation (IVF) [120,121], these IVF associated with early repolarization was also called early repolarization syndrome (ERS). Mutations in the LTCC genes, including *CACNA1C* and *CACNB2b*, have been found to associate with both diseases. Burashnikov et al. reported *CACNB2b* p.A73V mutation was associated with IVF; and *CACNB2b* p.S160T or p.R571C, *CACNA2D1* p.S956T, and *CACNA1C* p.E850 deletion had possible roles in ERS [102]. The glutamic acid at 850 position is located within the PEST domain of Ca_V_α_1_ II-III loop. The mutations in the PEST domain are proved to affect the surface membrane expression of Ca_V_1.2 channels [84]. Functionally, p.E850 deletion in *CACNA1C* is found to lead to an almost complete loss-of-function in *I*_Ca_ [90]. Thus, the p.E850 deletion might decrease the surface expression of LTCC presumably because of an aberrant protein degradation, which induces a dramatic reduction of *I*_Ca_.

### Bradycardia and sinoatrial arrhythmia

Autorhythmicity of sinoatrial nodal pacemaker cells results from the slow auto-depolarization as a result of Ca_V_1.3 calcium influx [122,123]. The inactivated Ca_V_1.3 channels in mice induced strong reduction of calcium current in pacemaker cells and profoundly affected its pacemaking effect, which showed predominant sinoatrial nodal dysfunction [124]. With the inactivated Ca_V_1.3 calcium currents, both sinoatrial arrhythmia and bradycardia have been observed [23,125]. A homozygous 3-bp insertion in *CACNA1D*, inducing p.G403_404ins, was first screened from a Pakistani family with pronounced bradycardia. This p.G403_404ins, located at alternative spliced exon 8b of Ca_V_α_1D_, resulted in nonconducting Ca_V_1.3 channels [126], which may explain the phenotype of bradycardia.

## Challenges and future diagnostic or therapeutic approaches

Mosaicism implies that in an organism from a single zygote that is the presence of genetically distinct cell line [127]. Taken TS as an example, the p.G406R mutation in exon 8 could be detected in the oral mucosa sample but not in the blood sample of the patients’ mother [61], whereas in the case of a Chinese girl with a typical TS phenotype, the G406R mutation also expressed in her father’s oral mucosa, sperm and white blood cell [128]. Additionally, cardiac arrhythmias also have phenotypic heterogeneity. The genotype-negative or even phenotype-negative LQT8 patients had been identified several novel mutations in *CACNA1C* [82,88]. Hence, careful screening of parental tissue in family with incidence of congenital cardiac arrhythmias, prenatal DNA screening, and monitoring of the fetal echocardiogram are extremely significant for the congenital cardiac arrhythmia. Remarkably, whole-genome or whole-exome sequencing [129] may be necessary for the identification of potential mutations of LTCC in cardiac arrhythmias.

LTCC has many variants because of transcriptional and post-transcriptional modification, which is complicate and may be the future challenges of the field. For example, alternative splicing, one of most important mechanisms of post-transcriptional modification [130,131], generates more than 20 alternative spliced exons in α_1C_ mRNA, which forms many variants of α_1C_ [14,60,132]. It has been indicated the mutations in these alternative spliced exons of LTCC can also attribute to the abnormal cardiac excitation [61,62,126]. Therefore, it is noteworthy to screen the potential mutations in these alternative spliced exons of LTCC in the patients with cardiac arrhythmias.

To date, most functional studies utilized heterologous systems expressing WT or mutant calcium channels in the cultured cells, which do not fully recapitulate the phenotypes of cardiomyocytes. With the advent of iPSC, the concept of cellular reprogramming has been revolutionized, which makes producing unique human iPSC from somatic cells possible [133], then induced to specific cells. TS patient-specific iPSC-derived cardiomyocyte (iPSC-CM) carrying a missense mutation p.G406R in the Ca_V_1.2 had been generated by traditional method or genetically encoded fluorescent indicators, which increases the inward calcium currents leading to prolonged QT interval [72,134]. Recently, genetic mutations could be directly introduced into generic embryonic stem cell or non-diseased iPSCs by genome editing techniques, to produce the phenotypes of cardiac arrhythmia [135], providing an exciting approach to drug screening in the iPSC-CM-based disease model. However, iPSC techniques have several limitations when being used as a treatment strategy for the diseased heart, e.g. the stem cell-derived cardiomyocytes being cocultured with native cardiomyocytes showed an slow conduction velocity [136] and inadequate excitation contraction coupling [137]. Nevertheless, the iPSC approach could potentially be a powerful tool for diagnosis and prognosis of cardiac arrhythmias and provide a robust assay for developing new drugs to treat these diseases [138–140]. Moreover, isogenetic control of iPSCs provides a method to reveal the pathogenic mechanism underlying the specific disease phenotype. In brief, the iPSC-based technology will play a central role in specific aspects of translational medicine [141].

It has been understood that major LTCC mutation-associated cardiac arrhythmias are attributed to the over-activities of calcium channel functions, which are due to the gain-of-function mutations in LTCC. However, the treatment of cardiac arrhythmias by calcium channel blockers meets a lot of challenges, e.g. limited range of available choices, and insufficient efficacy [2,142]. Moreover, the LTCC channel blockers, which have been used in clinic for decades, have none or very little selectivity for the different LTCC variants to some extent. At the same time, these drugs induce a lot of adverse effect risks because of off-target effects. Therefore, developing highly selective calcium channel blockers targeting different variants of LTCC, or even mutant LTCC will be much attractive in the management of these mutation-associated cardiac arrhythmias.

## Conclusions

Taken together, the mutations of the LTCC genes can cause different cardiac arrhythmias, including LQTS, TS, BrS, SQTS and other cardiac arrhythmias, which are critically associated with increased risks of cardiovascular diseases, syncope and even SCD. The mechanisms how these mutations cause the distinct cardiac arrhythmias are dependent on their different roles in the channel functions (structure-function mechanisms), thus more efforts on the crystal structure analysis of the LTCC are appreciated to understand these mutation-associated cardiac arrhythmias. Nevertheless, there are some challenges in the detection, diagnosis and treatment of cardiac arrhythmias at present, the future studies will be necessary to get a better understanding of the roles of LTCC in the context of cardiac arrhythmias, which will be much valuable for the diagnosis and even management of these diseases.

## References

[1] Almanac LR. Cardiac arrhythmias and pacing. Heart. 2013;99(19):1398–1407. PMID: 23906730. 23906730 10.1136/heartjnl-2013-304592

[2] Nattel S, Andrade J, Macle L, et al New directions in cardiac arrhythmia management: present challenges and future solutions. Can J Cardiol. 2014;30(12Suppl):S420–S30. PMID: 25432137. 25432137 10.1016/j.cjca.2014.09.027

[3] Bokil NJ, Baisden JM, Radford DJ, et al Molecular genetics of long QT syndrome. Mol Genet Metab. 2010;101(1):1–8. PMID: 20594883. 20594883 10.1016/j.ymgme.2010.05.011

[4] Hedley PL, Jorgensen P, Schlamowitz S, et al The genetic basis of long QT and short QT syndromes: a mutation update. Hum Mutat. 2009;30(11):1486–1511. PMID: 19862833. 19862833 10.1002/humu.21106

[5] Liao P, Soong TW CaV1.2 channelopathies: from arrhythmias to autism, bipolar disorder, and immunodeficiency. Pflugers Arch. 2010;460(2):353–359. PMID: 19916019. 19916019 10.1007/s00424-009-0753-0

[6] Catterall WA Structure and regulation of voltage-gated Ca2+ channels. Annu Rev Cell Dev Biol. 2000;16:521–555. PMID: 11031246. 11031246 10.1146/annurev.cellbio.16.1.521

[7] Schultz D, Mikala G, Yatani A, et al Cloning, chromosomal localization, and functional expression of the alpha 1 subunit of the L-type voltage-dependent calcium channel from normal human heart. Proc Natl Acad Sci U S A. 1993;90(13):6228–6232. PMID: 8392192. 8392192 10.1073/pnas.90.13.6228 PMC46901

[8] Dolphin AC Calcium channel diversity: multiple roles of calcium channel subunits. Curr Opin Neurobiol. 2009;19(3):237–244. PMID: 19559597. 19559597 10.1016/j.conb.2009.06.006

[9] Catterall WA Signaling complexes of voltage-gated sodium and calcium channels. Neurosci Lett. 2010;486(2):107–116. PMID: 20816922. 20816922 10.1016/j.neulet.2010.08.085 PMC3433163

[10] Catterall WA Voltage-gated calcium channels. Cold Spring Harb Perspect Biol. 2011;3(8):a003947 PMID: 21746798. 21746798 10.1101/cshperspect.a003947 PMC3140680

[11] Seisenberger C, Specht V, Welling A, et al Functional embryonic cardiomyocytes after disruption of the L-type alpha1C (Cav1.2) calcium channel gene in the mouse. J Biol Chem. 2000;275(50):39193–39199. PMID: 10973973. 10973973 10.1074/jbc.M006467200

[12] Moosmang S, Schulla V, Welling A, et al Dominant role of smooth muscle L-type calcium channel Cav1.2 for blood pressure regulation. EMBO J. 2003;22(22):6027–6034. PMID: 14609949. 14609949 10.1093/emboj/cdg583 PMC275441

[13] Hu Z, Lian MC, Soong TW Alternative Splicing of L-type CaV1.2 Calcium Channels: implications in Cardiovascular Diseases. Genes. 2017;8(12):344 PMID: 29186814. 10.3390/genes8120344 PMC5748662 29186814

[14] Liao P, Yong TF, Lian MC, et al Splicing for alternative structures of Cav1.2 Ca2+ channels in cardiac and smooth muscles. Cardiovasc Res. 2005;68(2):197–203. PMID: 16051206. 16051206 10.1016/j.cardiores.2005.06.024

[15] Zhou Y, Fan J, Zhu H, et al Aberrant splicing induced by dysregulated rbfox2 produces enhanced function of CaV1.2 calcium channel and vascular myogenic tone in hypertension. Hypertension. 2017;70(6):1183–1192. PMID: 28993448. 28993448 10.1161/HYPERTENSIONAHA.117.09301

[16] Wang J, Li G, Yu D, et al Characterization of CaV1.2 exon 33 heterozygous knockout mice and negative correlation between Rbfox1 and CaV1.2 exon 33 expressions in human heart failure. Channels. 2018;12(1):51–57. PMID: 28949795. 28949795 10.1080/19336950.2017.1381805 PMC5774182

[17] Li G, Wang J, Liao P, et al Exclusion of alternative exon 33 of CaV1.2 calcium channels in heart is proarrhythmogenic. Proc Natl Acad Sci U S A. 2017;114(21):E4288–E4295. PMID: 28490495. 28490495 10.1073/pnas.1617205114 PMC5448171

[18] Zhang Q, Timofeyev V, Qiu H, et al Expression and roles of Cav1.3 (alpha1D) L-type Ca(2)+ channel in atrioventricular node automaticity. J Mol Cell Cardiol. 2011;50(1):194–202. PMID: 20951705. 20951705 10.1016/j.yjmcc.2010.10.002 PMC3680510

[19] Brandt A, Striessnig J, Moser T CaV1.3 channels are essential for development and presynaptic activity of cochlear inner hair cells. J Neurosci. 2003;23(34):10832–10840. PMID: 14645476. 14645476 10.1523/JNEUROSCI.23-34-10832.2003 PMC6740966

[20] Matthes J, Yildirim L, Wietzorrek G, et al Disturbed atrio-ventricular conduction and normal contractile function in isolated hearts from Cav1.3-knockout mice. Naunyn Schmiedebergs Arch Pharmacol. 2004;369(6):554–562. PMID: 15146309. 15146309 10.1007/s00210-004-0940-7

[21] Green GE, Khan KM, Beisel DW, et al Calcium channel subunits in the mouse cochlea. J Neurochem. 1996;67(1):37–45. PMID: 8667015. 8667015 10.1046/j.1471-4159.1996.67010037.x

[22] Dou H, Vazquez AE, Namkung Y, et al Null mutation of alpha1D Ca2+ channel gene results in deafness but no vestibular defect in mice. J Assoc Res Otolaryngol. 2004;5(2):215–226. PMID: 15357422. 15357422 10.1007/s10162-003-4020-3 PMC2538408

[23] Platzer J, Engel J, Schrott-Fischer A, et al Congenital deafness and sinoatrial node dysfunction in mice lacking class D L-type Ca2+ channels. Cell. 2000;102(1):89–97. S0092-8674(00)00013-1 PMID: 10929716. 10929716 10.1016/s0092-8674(00)00013-1

[24] Van Petegem F, Clark KA, Chatelain FC, et al Structure of a complex between a voltage-gated calcium channel beta-subunit and an alpha-subunit domain. Nature. 2004;429(6992):671–675. PMID: 15141227. 15141227 10.1038/nature02588 PMC3076333

[25] Lao QZ, Kobrinsky E, Harry JB, et al New determinant for the CaVbeta2 subunit modulation of the CaV1.2 calcium channel. J Biol Chem. 2008;283(23):15577–15588. PMID: 18411278. 18411278 10.1074/jbc.M802035200 PMC2414265

[26] Cornet V, Bichet D, Sandoz G, et al Multiple determinants in voltage-dependent P/Q calcium channels control their retention in the endoplasmic reticulum. Eur J Neurosci. 2002;16(5):883–895. PMID: 12372025. 12372025 10.1046/j.1460-9568.2002.02168.x

[27] Castellano A, Perez-Reyes E Molecular diversity of Ca2+ channel beta subunits. Biochem Soc Trans. 1994;22(2):483–488. PMID: 7958351. 7958351 10.1042/bst0220483

[28] Walker D, De Waard M Subunit interaction sites in voltage-dependent Ca2+ channels: role in channel function. Trends Neurosci. 1998;21(4):148–154. S0166-2236(97)01200-9 PMID: 9554724. 9554724 10.1016/s0166-2236(97)01200-9

[29] Perez-Reyes E, Castellano A, Kim HS, et al Cloning and expression of a cardiac/brain beta subunit of the L-type calcium channel. J Biol Chem. 1992;267(3):1792–1797. PMID: 1370480. 1370480

[30] Takahashi SX, Mittman S, Colecraft HM Distinctive modulatory effects of five human auxiliary beta2 subunit splice variants on L-type calcium channel gating. Biophys J. 2003;84(5):3007–3021. S0006-3495(03)70027-7 PMID: 12719232. 12719232 10.1016/S0006-3495(03)70027-7 PMC1302863

[31] Buraei Z, Yang J The ss subunit of voltage-gated Ca2+ channels. Physiol Rev. 2010;90(4):1461–1506. PMID: 20959621. 20959621 10.1152/physrev.00057.2009 PMC4353500

[32] De Jongh KS, Warner C, Catterall WA Subunits of purified calcium channels. Alpha 2 and delta are encoded by the same gene. J Biol Chem. 1990;265(25):14738–14741. PMID: 2168391. 2168391

[33] Klugbauer N, Lacinova L, Marais E, et al Molecular diversity of the calcium channel alpha2delta subunit. J Neurosci. 1999;19(2):684–691. PMID: 9880589. 9880589 10.1523/JNEUROSCI.19-02-00684.1999 PMC6782206

[34] Tuluc P, Kern G, Obermair GJ, et al Computer modeling of siRNA knockdown effects indicates an essential role of the Ca2+ channel alpha2delta-1 subunit in cardiac excitation-contraction coupling. Proc Natl Acad Sci U S A. 2007;104(26):11091–11096. PMID: 17563358. 17563358 10.1073/pnas.0700577104 PMC1904133

[35] Barclay J, Balaguero N, Mione M, et al Ducky mouse phenotype of epilepsy and ataxia is associated with mutations in the Cacna2d2 gene and decreased calcium channel current in cerebellar Purkinje cells. J Neurosci. 2001;21(16):6095–6104. PMID: 11487633. 11487633 10.1523/JNEUROSCI.21-16-06095.2001 PMC6763162

[36] Qin N, Yagel S, Momplaisir ML, et al Molecular cloning and characterization of the human voltage-gated calcium channel alpha(2)delta-4 subunit. Mol Pharmacol. 2002;62(3):485–496. PMID: 12181424. 12181424 10.1124/mol.62.3.485

[37] Wycisk KA, Zeitz C, Feil S, et al Mutation in the auxiliary calcium-channel subunit CACNA2D4 causes autosomal recessive cone dystrophy. Am J Hum Genet. 2006;79(5):973–977. PMID: 17033974. 17033974 10.1086/508944 PMC1698577

[38] Whittaker CA, Hynes RO Distribution and evolution of von Willebrand/integrin A domains: widely dispersed domains with roles in cell adhesion and elsewhere. Mol Biol Cell. 2002;13(10):3369–3387. PMID: 12388743. 12388743 10.1091/mbc.E02-05-0259 PMC129952

[39] Bannister JP, Bulley S, Narayanan D, et al Transcriptional upregulation of alpha2delta-1 elevates arterial smooth muscle cell voltage-dependent Ca2+ channel surface expression and cerebrovascular constriction in genetic hypertension. Hypertension. 2012;60(4):1006–1015. PMID: 22949532. 22949532 10.1161/HYPERTENSIONAHA.112.199661 PMC3632309

[40] Bannister JP, Adebiyi A, Zhao G, et al Smooth muscle cell alpha2delta-1 subunits are essential for vasoregulation by CaV1.2 channels. Circ Res. 2009;105(10):948–955. PMID: 19797702. 19797702 10.1161/CIRCRESAHA.109.203620 PMC2783418

[41] Hatano S, Yamashita T, Sekiguchi A, et al Molecular and electrophysiological differences in the L-type Ca2+ channel of the atrium and ventricle of rat hearts. Circ J. 2006;70(5):610–614. JST.JSTAGE/circj/70.610 PMID: 16636499. 16636499 10.1253/circj.70.610

[42] Fuller-Bicer GA, Varadi G, Koch SE, et al Targeted disruption of the voltage-dependent calcium channel alpha2/delta-1-subunit. Am J Physiol Heart Circ Physiol. 2009;297(1):H117–H24. PMID: 19429829. 19429829 10.1152/ajpheart.00122.2009 PMC2711723

[43] Bosse E, Regulla S, Biel M, et al The cDNA and deduced amino acid sequence of the gamma subunit of the L-type calcium channel from rabbit skeletal muscle. FEBS Lett. 1990;267(1):153–156. 0014-5793(90)80312-7 PMID: 2163895. 2163895 10.1016/0014-5793(90)80312-7

[44] Jay SD, Ellis SB, McCue AF, et al Primary structure of the gamma subunit of the DHP-sensitive calcium channel from skeletal muscle. Science. 1990;248(4954):490–492. PMID: 2158672. 2158672 10.1126/science.2158672

[45] Chen RS, Deng TC, Garcia T, et al Calcium channel gamma subunits: a functionally diverse protein family. Cell Biochem Biophys. 2007;47(2):178–186. CBB:47:2:178 PMID: 17652770. 17652770 10.1007/s12013-007-0002-0

[46] Yang L, Katchman A, Morrow JP, et al Cardiac L-type calcium channel (Cav1.2) associates with gamma subunits. FASEB J. 2011;25(3):928–936. PMID: 21127204. 21127204 10.1096/fj.10-172353 PMC3042847

[47] Freise D, Held B, Wissenbach U, et al Absence of the gamma subunit of the skeletal muscle dihydropyridine receptor increases L-type Ca2+ currents and alters channel inactivation properties. J Biol Chem. 2000;275(19):14476–14481. PMID: 10799530. 10799530 10.1074/jbc.275.19.14476

[48] Klugbauer N, Dai S, Specht V, et al A family of gamma-like calcium channel subunits. FEBS Lett. 2000;470(2):189–197. PMID: 10734232. 10734232 10.1016/s0014-5793(00)01306-5

[49] Schwartz PJ, Stramba-Badiale M, Crotti L, et al Prevalence of the congenital long-QT syndrome. Circulation. 2009;120(18):1761–1767. PMID: 19841298. 19841298 10.1161/CIRCULATIONAHA.109.863209 PMC2784143

[50] Moss AJ, Kass RS Long QT syndrome: from channels to cardiac arrhythmias. J Clin Invest. 2005;115(8):2018–2024. PMID: 16075042. 16075042 10.1172/JCI25537 PMC1180552

[51] Kannankeril PJ, Roden DM Drug-induced long QT and torsade de pointes: recent advances. Curr Opin Cardiol. 2007;22(1):39–43. PMID: 17143043. 17143043 10.1097/HCO.0b013e32801129eb

[52] Saffitz JE Structural heart disease, SCN5A gene mutations, and Brugada syndrome: a complex menage a trois. Circulation. 2005;112(24):3672–3674. PMID: 16344397. 16344397 10.1161/CIRCULATIONAHA.105.587147

[53] Mahida S, Hogarth AJ, Cowan C, et al Genetics of congenital and drug-induced long QT syndromes: current evidence and future research perspectives. J Interv Card Electrophysiol. 2013;37(1):9–19. PMID: 23515882. 23515882 10.1007/s10840-013-9779-5

[54] Schwartz PJ, Ackerman MJ, George AL Jr, et al Impact of genetics on the clinical management of channelopathies. J Am Coll Cardiol. 2013;62(3):169–180. PMID: 23684683. 23684683 10.1016/j.jacc.2013.04.044 PMC3710520

[55] Marks ML, Trippel DL, Keating MT Long QT syndrome associated with syndactyly identified in females. Am J Cardiol. 1995;76(10):744–745. S0002-9149(99)80216-1 PMID: 7572644. 7572644 10.1016/s0002-9149(99)80216-1

[56] Marks ML, Whisler SL, Clericuzio C, et al A new form of long QT syndrome associated with syndactyly. J Am Coll Cardiol. 1995;25(1):59–64. 0735-1097(94)00318-K PMID: 7798527. 7798527 10.1016/0735-1097(94)00318-k

[57] Reichenbach H, Meister EM, Theile H [The heart-hand syndrome. A new variant of disorders of heart conduction and syndactylia including osseous changes in hands and feet]. Kinderarztl Prax. 1992;60(2):54–56. PMID: 1318983. 1318983

[58] Corona-Rivera JR, Barrios-Prieto E, Nieto-Garcia R, et al Unusual retrospective prenatal findings in a male newborn with Timothy syndrome type 1. Eur J Med Genet. 2015;58(6–7):332–335. PMID: 25882468. 25882468 10.1016/j.ejmg.2015.04.001

[59] La-A-Njoe SM, Wilde AA, van Erven L, et al Syndactyly and long QT syndrome (CaV1.2 missense mutation G406R) is associated with hypertrophic cardiomyopathy. Heart Rhythm. 2005;2(12):1365–1368. PMID: 16360093. 16360093 10.1016/j.hrthm.2005.08.024

[60] Tang ZZ, Lian MC, Lu S, et al Transcript scanning reveals novel and extensive splice variations in human l-type voltage-gated calcium channel, Cav1.2 alpha1 subunit. J Biol Chem. 2004;279(43):44335–44343. PMID: 15299022. 15299022 10.1074/jbc.M407023200

[61] Splawski I, Timothy KW, Sharpe LM, et al Ca(V)1.2 calcium channel dysfunction causes a multisystem disorder including arrhythmia and autism. Cell. 2004;119(1):19–31. PMID: 15454078. 15454078 10.1016/j.cell.2004.09.011

[62] Splawski I, Timothy KW, Decher N, et al Severe arrhythmia disorder caused by cardiac L-type calcium channel mutations. Proc Natl Acad Sci U S A. 2005;102(23):8089–8096. PMID: 15863612. 15863612 10.1073/pnas.0502506102 PMC1149428

[63] Stotz SC, Zamponi GW Identification of inactivation determinants in the domain IIS6 region of high voltage-activated calcium channels. J Biol Chem. 2001;276(35):33001–33010. PMID: 11402052. 11402052 10.1074/jbc.M104387200

[64] Findlay I Physiological modulation of inactivation in L-type Ca2+ channels: one switch. J Physiol. 2004;554(Pt2):275–283. PMID: 12824441. 12824441 10.1113/jphysiol.2003.047902 PMC1664755

[65] Cens T, Rousset M, Leyris JP, et al Voltage- and calcium-dependent inactivation in high voltage-gated Ca(2+) channels. Prog Biophys Mol Biol. 2006;90(1–3):104–117. PMID: 16038964. 16038964 10.1016/j.pbiomolbio.2005.05.013

[66] Barrett CF, Tsien RW The Timothy syndrome mutation differentially affects voltage- and calcium-dependent inactivation of CaV1.2 L-type calcium channels. Proc Natl Acad Sci U S A. 2008;105(6):2157–2162. PMID: 18250309. 18250309 10.1073/pnas.0710501105 PMC2538892

[67] Raybaud A, Dodier Y, Bissonnette P, et al The role of the GX9GX3G motif in the gating of high voltage-activated Ca2+ channels. J Biol Chem. 2006;281(51):39424–39436. PMID: 17038321. 17038321 10.1074/jbc.M607405200

[68] Depil K, Beyl S, Stary-Weinzinger A, et al Timothy mutation disrupts the link between activation and inactivation in Ca(V)1.2 protein. J Biol Chem. 2011;286(36):31557–31564. PMID: 21685391. 21685391 10.1074/jbc.M111.255273 PMC3173108

[69] Stotz SC, Jarvis SE, Zamponi GW Functional roles of cytoplasmic loops and pore lining transmembrane helices in the voltage-dependent inactivation of HVA calcium channels. J Physiol. 2004;554(Pt2):263–273. PMID: 12815185. 12815185 10.1113/jphysiol.2003.047068 PMC1664770

[70] Huang H, Wang J, Soong TW Alternative exon effect on phenotype of Cav1.2 channelopathy: implications in Timothy syndrome In: Weiss N, Koschak A, editors. Pathologies of Calcium Channels. Berlin, Heidelberg: Springer; 2014.

[71] Cheng EP, Yuan C, Navedo MF, et al Restoration of normal L-type Ca2+ channel function during Timothy syndrome by ablation of an anchoring protein. Circ Res. 2011;109(3):255–261. PMID: 21700933. 21700933 10.1161/CIRCRESAHA.111.248252 PMC3151468

[72] Yazawa M, Hsueh B, Jia X, et al Using induced pluripotent stem cells to investigate cardiac phenotypes in Timothy syndrome. Nature. 2011;471(7337):230–234. PMID: 21307850. 21307850 10.1038/nature09855 PMC3077925

[73] Pasca SP, Portmann T, Voineagu I, et al Using iPSC-derived neurons to uncover cellular phenotypes associated with Timothy syndrome. Nat Med. 2011;17(12):1657–1662. PMID: 22120178. 22120178 10.1038/nm.2576 PMC3517299

[74] Yarotskyy V, Elmslie KS Roscovitine, a cyclin-dependent kinase inhibitor, affects several gating mechanisms to inhibit cardiac L-type (Ca(V)1.2) calcium channels. Br J Pharmacol. 2007;152(3):386–395. PMID: 17700718. 17700718 10.1038/sj.bjp.0707414 PMC2042960

[75] Yarotskyy V, Gao G, Peterson BZ, et al The Timothy syndrome mutation of cardiac CaV1.2 (L-type) channels: multiple altered gating mechanisms and pharmacological restoration of inactivation. J Physiol. 2009;587(Pt3):551–565. PMID: 19074970. 19074970 10.1113/jphysiol.2008.161737 PMC2670080

[76] Drum BM, Dixon RE, Yuan C, et al Cellular mechanisms of ventricular arrhythmias in a mouse model of Timothy syndrome (long QT syndrome 8). J Mol Cell Cardiol. 2014;66:63–71. PMID: 24215710. 24215710 10.1016/j.yjmcc.2013.10.021 PMC3903114

[77] Ramachandran KV, Hennessey JA, Barnett AS, et al Calcium influx through L-type CaV1.2 Ca2+ channels regulates mandibular development. J Clin Invest. 2013;123(4):1638–1646. PMID: 23549079. 23549079 10.1172/JCI66903 PMC3613930

[78] Hiippala A, Tallila J, Myllykangas S, et al Expanding the phenotype of Timothy syndrome type 2: an adolescent with ventricular fibrillation but normal development. Am J Med Genet A. 2015;167A(3):629–634. PMID: 25691416. 25691416 10.1002/ajmg.a.36924

[79] Bader PL, Faizi M, Kim LH, et al Mouse model of Timothy syndrome recapitulates triad of autistic traits. Proc Natl Acad Sci U S A. 2011;108(37):15432–15437. PMID: 21878566. 21878566 10.1073/pnas.1112667108 PMC3174658

[80] Gillis J, Burashnikov E, Antzelevitch C, et al Long QT, syndactyly, joint contractures, stroke and novel CACNA1C mutation: expanding the spectrum of Timothy syndrome. Am J Med Genet A. 2012;158A(1):182–187. PMID: 22106044. 22106044 10.1002/ajmg.a.34355 PMC3319791

[81] Boczek NJ, Miller EM, Ye D, et al Novel Timothy syndrome mutation leading to increase in CACNA1C window current. Heart Rhythm. 2015;12(1):211–219. PMID: 25260352. 25260352 10.1016/j.hrthm.2014.09.051 PMC4907369

[82] Wemhoner K, Friedrich C, Stallmeyer B, et al Gain-of-function mutations in the calcium channel CACNA1C (Cav1.2) cause non-syndromic long-QT but not Timothy syndrome. J Mol Cell Cardiol. 2015;80:186–195. PMID: 25633834. 25633834 10.1016/j.yjmcc.2015.01.002

[83] Boczek NJ, Ye D, Jin F, et al Identification and functional characterization of a novel CACNA1C-Mediated cardiac disorder characterized by prolonged QT intervals with hypertrophic cardiomyopathy, congenital heart defects, and sudden cardiac death. Circ Arrhythm Electrophysiol. 2015;8(5):1122–1132. PMID: 26253506. 26253506 10.1161/CIRCEP.115.002745 PMC5094060

[84] Boczek NJ, Best JM, Tester DJ, et al Exome sequencing and systems biology converge to identify novel mutations in the L-type calcium channel, CACNA1C, linked to autosomal dominant long QT syndrome. Circ Cardiovasc Genet. 2013;6(3):279–289. PMID: 23677916. 23677916 10.1161/CIRCGENETICS.113.000138 PMC3760222

[85] Rechsteiner M, Rogers SW PEST sequences and regulation by proteolysis. Trends Biochem Sci. 1996;21(7):267–271. S0968-0004(96)10031-1 PMID: 8755249. 8755249

[86] Wang Y, Deng X, Mancarella S, et al The calcium store sensor, STIM1, reciprocally controls Orai and CaV1.2 channels. Science. 2010;330(6000):105–109. PMID: 20929813. 20929813 10.1126/science.1191086 PMC3601900

[87] Park CY, Shcheglovitov A, Dolmetsch R The CRAC channel activator STIM1 binds and inhibits L-type voltage-gated calcium channels. Science. 2010;330(6000):101–105. PMID: 20929812. 20929812 10.1126/science.1191027

[88] Fukuyama M, Wang Q, Kato K, et al Long QT syndrome type 8: novel CACNA1C mutations causing QT prolongation and variant phenotypes. Europace. 2014;16(12):1828–1837. PMID: 24728418. 24728418 10.1093/europace/euu063

[89] Landstrom AP, Boczek NJ, Ye D, et al Novel long QT syndrome-associated missense mutation, L762F, in CACNA1C-encoded L-type calcium channel imparts a slower inactivation tau and increased sustained and window current. Int J Cardiol. 2016;220:290–298. PMID: 27390944. 27390944 10.1016/j.ijcard.2016.06.081 PMC6311393

[90] Sutphin BS, Boczek NJ, Barajas-Martinez H, et al Molecular and functional characterization of rare CACNA1C variants in sudden unexplained death in the young. Congenit Heart Dis. 2016;11(6):683–692. PMID: 27218670. 27218670 10.1111/chd.12371

[91] Brugada P, Brugada J Right bundle branch block, persistent ST segment elevation and sudden cardiac death: a distinct clinical and electrocardiographic syndrome. A multicenter report. J Am Coll Cardiol. 1992;20(6):1391–1396. 0735-1097(92)90253-J PMID: 1309182. 1309182 10.1016/0735-1097(92)90253-j

[92] Huang MH, Marcus FI Idiopathic Brugada-type electrocardiographic pattern in an octogenarian. J Electrocardiol. 2004;37(2):109–111. S0022073604000147 PMID: 15127377. 15127377 10.1016/j.jelectrocard.2004.01.007

[93] Antzelevitch C, Brugada P, Borggrefe M, et al Brugada syndrome: report of the second consensus conference: endorsed by the Heart Rhythm Society and the European Heart Rhythm Association. Circulation. 2005;111(5):659–670. PMID: 15655131. 15655131 10.1161/01.CIR.0000152479.54298.51

[94] Antzelevitch C Brugada syndrome. Pacing Clin Electrophysiol. 2006;29(10):1130–1159. PMID: 17038146. 17038146 10.1111/j.1540-8159.2006.00507.x PMC1978482

[95] Di Diego JM, Cordeiro JM, Goodrow RJ, et al Ionic and cellular basis for the predominance of the Brugada syndrome phenotype in males. Circulation. 2002;106(15):2004–2011. PMID: 12370227. 12370227 10.1161/01.cir.0000032002.22105.7a

[96] Nielsen MW, Holst AG, Olesen SP, et al The genetic component of Brugada syndrome. Front Physiol. 2013;4:179 PMID: 23874304. 23874304 10.3389/fphys.2013.00179 PMC3710955

[97] Brugada R, Campuzano O, Brugada P, et al Brugada Syndrome. 1993 NBK1517 [bookaccession] In: Adam MP, Ardinger HH, Pagon RA, Wallace SE, Bean LJH, Stephens K, Amemiya A, editors. GeneReviews. Seattle, WA. PMID: 20301690.

[98] Antzelevitch C, Pollevick GD, Cordeiro JM, et al Loss-of-function mutations in the cardiac calcium channel underlie a new clinical entity characterized by ST-segment elevation, short QT intervals, and sudden cardiac death. Circulation. 2007;115(4):442–449. PMID: 17224476. 17224476 10.1161/CIRCULATIONAHA.106.668392 PMC1952683

[99] Simms BA, Souza IA, Zamponi GW Effect of the Brugada syndrome mutation A39V on calmodulin regulation of Cav1.2 channels. Mol Brain. 2014;7:34 PMID: 24775099. 24775099 10.1186/1756-6606-7-34 PMC4012176

[100] Simms BA, Zamponi GW The Brugada syndrome mutation A39V does not affect surface expression of neuronal rat Cav1.2 channels. Mol Brain. 2012;5:9 PMID: 22385640. 22385640 10.1186/1756-6606-5-9 PMC3307476

[101] Fukuyama M, Ohno S, Wang Q, et al Nonsense-mediated mRNA decay due to a CACNA1C splicing mutation in a patient with Brugada syndrome. Heart Rhythm. 2014;11(4):629–634. PMID: 24321233. 24321233 10.1016/j.hrthm.2013.12.011

[102] Burashnikov E, Pfeiffer R, Barajas-Martinez H, et al Mutations in the cardiac L-type calcium channel associated with inherited J-wave syndromes and sudden cardiac death. Heart Rhythm. 2010;7(12):1872–1882. PMID: 20817017. 20817017 10.1016/j.hrthm.2010.08.026 PMC2999985

[103] Simms B Brugada Syndrome and Voltage-Gated Calcium Channels In: Weiss N, Koschak A, editors. Pathologies of Calcium Channels. Berlin, Heidelberg: Springer; 2014.

[104] Cordeiro JM, Marieb M, Pfeiffer R, et al Accelerated inactivation of the L-type calcium current due to a mutation in CACNB2b underlies Brugada syndrome. J Mol Cell Cardiol. 2009;46(5):695–703. PMID: 19358333. 19358333 10.1016/j.yjmcc.2009.01.014 PMC2668128

[105] Hedley PL, Jorgensen P, Schlamowitz S, et al The genetic basis of Brugada syndrome: a mutation update. Hum Mutat. 2009;30(9):1256–1266. PMID: 19606473. 19606473 10.1002/humu.21066

[106] Crotti L, Marcou CA, Tester DJ, et al Spectrum and prevalence of mutations involving BrS1- through BrS12-susceptibility genes in a cohort of unrelated patients referred for Brugada syndrome genetic testing: implications for genetic testing. J Am Coll Cardiol. 2012;60(15):1410–1418. PMID: 22840528. 22840528 10.1016/j.jacc.2012.04.037 PMC3624764

[107] Risgaard B, Jabbari R, Refsgaard L, et al High prevalence of genetic variants previously associated with Brugada syndrome in new exome data. Clin Genet. 2013;84(5):489–495. PMID: 23414114. 23414114 10.1111/cge.12126

[108] Gussak I, Brugada P, Brugada J, et al Idiopathic short QT interval: a new clinical syndrome? Cardiology. 2000;94(2):99–102. PMID: 11173780. 11173780 10.1159/000047299

[109] Bjerregaard P, Gussak I Short QT syndrome. Ann Noninvasive Electrocardiol. 2005;10(4):436–440. PMID: 16255754. 16255754 10.1111/j.1542-474X.2005.00064.x PMC6932734

[110] McPate MJ, Witchel HJ, Hancox JC Short QT syndrome. Future Cardiol. 2006;2(3):293–301. PMID: 19804087. 19804087 10.2217/14796678.2.3.293

[111] Brugada R, Hong K, Dumaine R, et al Sudden death associated with short-QT syndrome linked to mutations in HERG. Circulation. 2004;109(1):30–35. PMID: 14676148. 14676148 10.1161/01.CIR.0000109482.92774.3A

[112] Bellocq C, van Ginneken AC, Bezzina CR, et al Mutation in the KCNQ1 gene leading to the short QT-interval syndrome. Circulation. 2004;109(20):2394–2397. PMID: 15159330. 15159330 10.1161/01.CIR.0000130409.72142.FE

[113] Giustetto C, Schimpf R, Mazzanti A, et al Long-term follow-up of patients with short QT syndrome. J Am Coll Cardiol. 2011;58(6):587–595. PMID: 21798421. 21798421 10.1016/j.jacc.2011.03.038

[114] Gollob MH, Redpath CJ, Roberts JD The short QT syndrome: proposed diagnostic criteria. J Am Coll Cardiol. 2011;57(7):802–812. PMID: 21310316. 21310316 10.1016/j.jacc.2010.09.048

[115] Mazzanti A, Kanthan A, Monteforte N, et al Novel insight into the natural history of short QT syndrome. J Am Coll Cardiol. 2014;63(13):1300–1308. PMID: 24291113. 24291113 10.1016/j.jacc.2013.09.078 PMC3988978

[116] Chen Y, Barajas-Martinez H, Zhu D, et al Novel trigenic CACNA1C/DES/MYPN mutations in a family of hypertrophic cardiomyopathy with early repolarization and short QT syndrome. J Transl Med. 2017;15(1):78 PMID: 28427417. 28427417 10.1186/s12967-017-1180-1 PMC5399316

[117] Templin C, Ghadri JR, Rougier JS, et al Identification of a novel loss-of-function calcium channel gene mutation in short QT syndrome (SQTS6). Eur Heart J. 2011;32(9):1077–1088. PMID: 21383000. 21383000 10.1093/eurheartj/ehr076 PMC3086900

[118] Kanter RJ, Pfeiffer R, Hu D, et al Brugada-like syndrome in infancy presenting with rapid ventricular tachycardia and intraventricular conduction delay. Circulation. 2012;125(1):14–22. PMID: 22090166. 22090166 10.1161/CIRCULATIONAHA.111.054007 PMC3519939

[119] Hu D, Barajas-Martinez H, Nesterenko VV, et al Dual variation in SCN5A and CACNB2b underlies the development of cardiac conduction disease without Brugada syndrome. Pacing Clin Electrophysiol. 2010;33(3):274–285. PMID: 20025708. 20025708 10.1111/j.1540-8159.2009.02642.x PMC2916871

[120] Aizawa Y, Tamura M, Chinushi M, et al Idiopathic ventricular fibrillation and bradycardia-dependent intraventricular block. Am Heart J. 1993;126(6):1473–1474. PMID: 8249808. 8249808 10.1016/0002-8703(93)90550-s

[121] Haissaguerre M, Derval N, Sacher F, et al Sudden cardiac arrest associated with early repolarization. N Engl J Med. 2008;358(19):2016–2023. PMID: 18463377. 18463377 10.1056/NEJMoa071968

[122] Gilmour RF Jr, Zipes DP Slow inward current and cardiac arrhythmias. Am J Cardiol. 1985;55(3):89B–101B. PMID: 2857519. 2857519 10.1016/0002-9149(85)90617-4

[123] Verheijck EE, van Ginneken AC, Wilders R, et al Contribution of L-type Ca2+ current to electrical activity in sinoatrial nodal myocytes of rabbits. Am J Physiol. 1999;276(3 Pt 2):H1064–H77. PMID: 10070093. 10070093 10.1152/ajpheart.1999.276.3.H1064

[124] Mangoni ME, Couette B, Bourinet E, et al Functional role of L-type Cav1.3 Ca2+ channels in cardiac pacemaker activity. Proc Natl Acad Sci U S A. 2003;100(9):5543–5548. PMID: 12700358. 12700358 10.1073/pnas.0935295100 PMC154381

[125] Zhang Z, Xu Y, Song H, et al Functional Roles of Ca(v)1.3 (alpha(1D)) calcium channel in sinoatrial nodes: insight gained using gene-targeted null mutant mice. Circ Res. 2002;90(9):981–987. PMID: 12016264. 12016264 10.1161/01.res.0000018003.14304.e2

[126] Baig SM, Koschak A, Lieb A, et al Loss of Ca(v)1.3 (CACNA1D) function in a human channelopathy with bradycardia and congenital deafness. Nat Neurosci. 2011;14(1):77–84. PMID: 21131953. 21131953 10.1038/nn.2694

[127] Youssoufian H, Pyeritz RE Mechanisms and consequences of somatic mosaicism in humans. Nat Rev Genet. 2002;3(10):748–758. PMID: 12360233. 12360233 10.1038/nrg906

[128] Gao Y, Xue X, Hu D, et al Inhibition of late sodium current by mexiletine: a novel pharmotherapeutical approach in timothy syndrome. Circ Arrhythm Electrophysiol. 2013;6(3):614–622. PMID: 23580742. 23580742 10.1161/CIRCEP.113.000092

[129] Frohler S, Kieslich M, Langnick C, et al Exome sequencing helped the fine diagnosis of two siblings afflicted with atypical Timothy syndrome (TS2). BMC Med Genet. 2014;15:48 PMID: 24773605. 24773605 10.1186/1471-2350-15-48 PMC4038115

[130] Wang ET, Sandberg R, Luo S, et al Alternative isoform regulation in human tissue transcriptomes. Nature. 2008;456(7221):470–476. PMID: 18978772. 18978772 10.1038/nature07509 PMC2593745

[131] Pan Q, Shai O, Lee LJ, et al Deep surveying of alternative splicing complexity in the human transcriptome by high-throughput sequencing. Nat Genet. 2008;40(12):1413–1415. PMID: 18978789. 18978789 10.1038/ng.259

[132] Tang ZZ, Hong X, Wang J, et al Signature combinatorial splicing profiles of rat cardiac- and smooth-muscle Cav1.2 channels with distinct biophysical properties. Cell Calcium. 2007;41(5):417–428. PMID: 16979758. 16979758 10.1016/j.ceca.2006.08.002

[133] Takahashi K, Yamanaka S Induction of pluripotent stem cells from mouse embryonic and adult fibroblast cultures by defined factors. Cell. 2006;126(4):663–676. PMID: 16904174. 16904174 10.1016/j.cell.2006.07.024

[134] Song L, Awari DW, Han EY, et al Dual optical recordings for action potentials and calcium handling in induced pluripotent stem cell models of cardiac arrhythmias using genetically encoded fluorescent indicators. Stem Cells Transl Med. 2015;4(5):468–475. PMID: 25769651. 25769651 10.5966/sctm.2014-0245 PMC4414223

[135] Wang Y, Liang P, Lan F, et al Genome editing of isogenic human induced pluripotent stem cells recapitulates long QT phenotype for drug testing. J Am Coll Cardiol. 2014;64(5):451–459. PMID: 25082577. 25082577 10.1016/j.jacc.2014.04.057 PMC4149735

[136] Kucera JP, Prudat Y, Marcu IC, et al Slow conduction in mixed cultured strands of primary ventricular cells and stem cell-derived cardiomyocytes. Front Cell Dev Biol. 2015;3:58 PMID: 26442264. 26442264 10.3389/fcell.2015.00058 PMC4585316

[137] Marcu IC, Illaste A, Heuking P, et al Functional Characterization and Comparison of Intercellular Communication in Stem Cell-Derived Cardiomyocytes. Stem Cells. 2015;33(7):2208–2218. PMID: 25968594. 25968594 10.1002/stem.2009

[138] Knollmann BC Induced pluripotent stem cell-derived cardiomyocytes: boutique science or valuable arrhythmia model? Circ Res. 2013;112(6):969–976. PMID: 23569106. 23569106 10.1161/CIRCRESAHA.112.300567 PMC3667201

[139] Terrenoire C, Wang K, Tung KW, et al Induced pluripotent stem cells used to reveal drug actions in a long QT syndrome family with complex genetics. J Gen Physiol. 2013;141(1):61–72. PMID: 23277474. 23277474 10.1085/jgp.201210899 PMC3536519

[140] Sinnecker D, Dirschinger RJ, Goedel A, et al Induced pluripotent stem cells in cardiovascular research. Rev Physiol Biochem Pharmacol. 2012;163:1–26. PMID: 22447279. 22447279 10.1007/112_2012_6

[141] Wu M, Chen G, Hu B Induced pluripotency for translational research. Genomics Proteomics Bioinf. 2013;11(5):288–293. PMID: 24056061. 10.1016/j.gpb.2013.08.001 PMC4357792 24056061

[142] Piccini JP, Zhao Y, Steinberg BA, et al Comparative effectiveness of pharmacotherapies for prevention of atrial fibrillation following coronary artery bypass surgery. Am J Cardiol. 2013;112(7):954–960. PMID: 23850476. 23850476 10.1016/j.amjcard.2013.05.029

